# Environmental pH modulation by pathogenic fungi as a strategy to conquer the host

**DOI:** 10.1371/journal.ppat.1006149

**Published:** 2017-02-23

**Authors:** Slavena Vylkova

**Affiliations:** 1 Septomics Research Center, Friedrich Schiller University, Jena, Germany; 2 Leibniz Institute for Natural Product Research and Infection Biology, Hans Knöll Institute, Jena, Germany; Geisel School of Medicine at Dartmouth, UNITED STATES

The ability of microorganisms to sense and adapt to changes in the environment is essential to their survival. This is particularly important for species with an intimate association with host organisms, such as pathogens, symbionts, and commensals. Host environments vary greatly in pH, ranging from highly acidic in the stomach (pH < 2) to mildly acidic on the skin and plant surfaces (6.5 < pH < 4.5), neutral in the blood (pH 7.4), and basic in parts of the intestine (pH < 8.5) [1–4], and fungi have developed multiple mechanisms to adapt to pH variations. This Pearl will focus on the ability of pathogenic fungi to respond to and actively modulate the host’s pH.

## Fungal adaptation to changes in ambient pH

In fungi, the adaptive responses induced by changes in ambient pH have been extensively studied in model organisms. For example, the response to weak acid stress has been characterized in *Saccharomyces cerevisiae* [5], while the role of the Pal/Rim alkaline response pathway, one of the most specialized and conserved signaling cascades in fungi, has been delineated in *S*. *cerevisiae*, *Aspergillus nidulans*, *Yarrowia lipolytica*, and several fungal pathogens [6–8]. The mechanisms of pH sensing and adaptation in fungi have been reviewed elsewhere [9–11].

## Fungal pathogens can modulate the pH of their host

Another aspect of pH regulation is the ability of microorganisms to actively modify the pH of their environment. Fungi can achieve this by secreting acids or alkali. The ability of fungi to secrete natural organic acids (such as butyrate, oxalate, malate, citrate, gluconate, and succinate) is well utilized in the industry, particularly with nonpathogenic *Aspergillus* sp. and *Rhizopus* sp. The magnitude of pH change depends on the nutrient availability, the organic acids being produced, and on the ability of the fungus to remove ammonium ions from ammonium sulfate salt or to excrete H^+^-ions as a byproduct of NH_4_^+^ assimilation [12, 13]. Acidifying fungi can also raise extremely low pH levels to a favorable level.

Certain pathogenic fungi acidify the environment as a strategy to damage host tissues. Many plant-necrotizing fungi secrete significant amounts of acid: *Sclerotinia sclerotiorum* and *Butrytis* sp. produce oxalic acid [14], while *Pennicilium* sp. and *Aspergillus* sp. secrete mainly gluconic and citric acids [15, 16]. The produced acids not only acidify the tissues but can also lower the activity of reactive oxygen species produced by the host [17]. Fusaric acid produced by *Fusarium oxysporum* acidifies plant surfaces and activates the membrane H^+^-ATPase, a pH-regulated process that leads to the expression of proteases and subsequent tissue invasion [18]. Similarly, the human pathogen *Candida albicans* acidifies the environment in a carbohydrate-dependent fashion, allowing production of aspartyl proteases, which are potent virulence factors [19].

## Ammonia as a key player in environmental alkalinization

Environmental alkalinization in fungi is common yet not a well-understood phenomenon. Often, this process is mediated by ammonia, a multifunctional biological molecule with diverse roles in eukaryotes. In fungi, it supports communication between colonies in *S*. *cerevisiae* [20], aging of surface-ripened cheeses by *Y*. *lipolytica* and *Debaryomyces hansenii* [21], and expression of pectin lyase, a key virulence factor in *Colletotrichum* sp. [22], among other functions.

**Fig 1:**
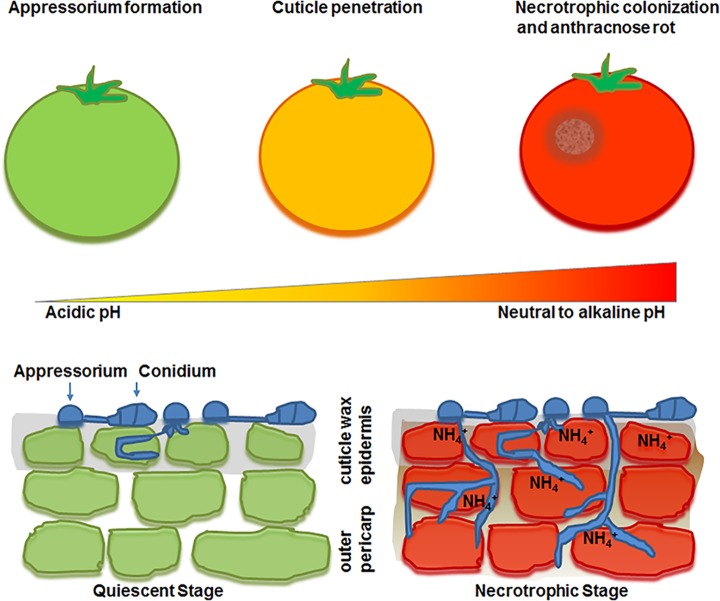
Modulation of host pH by the phytopathogen *Colletotrichum gloeosporioides* increases fungal virulence. *C*. *gloeosporioides* infects the tomato fruit in a process initiated upon attachment of the fungal conidia to the plant surface. During the quiescent stage of infection, fruit physiological factors such as nutrient availability, acidic pH, and surface waxes determine the rate of fungal growth and germination. As the fruit ripens, conidia germinate into a specialized structure, named appressorium, which eventually becomes melanized. Melanin alters the permeability of the plant cell wall, creating a hypertonic environment that allows the fungus to penetrate the host epidermis using turgor pressure. This process is accompanied by active metabolism of amino acids, such as glutamate and glutamine, and gradual environmental alkalization. The fungus transitions into the necrotrophic stage, characterized by a dramatic shift in fungal metabolism and activation of pathogenicity factors, such as proteases and lyases, resulting in anthracnose fruit rot.

Ammonia is generated either extracellularly or within the fungal cell as a byproduct of protein and amino acid catabolism, common nutrients in many host niches [23, 24]. Excess intracellular ammonia is secreted or exported from the cell or exported as urea and subsequently converted to NH_4_^+^ by secreted ureases. Accumulation of this highly basic compound in the immediate environment raises the pH. An excellent example of this process is found in the phytopathogen *Colletotrichum gloeosporioides*, a cause of anthracnose fruit rot. The fungus utilizes L-glutamate or glutamine to produce ammonia, which elevates the environmental pH of healthy fruit from 5.6 to 8.5. This results in the activation of fungal pathogenicity factors, such as production of pectate lyase, induction of appressorium formation during host penetration, and stimulation of host cell death mechanisms [22] (Fig 1). In other fungal species, the alkalinized environment also activates the expression of virulence traits: production of asexual spores and secretion of lytic enzymes in *Magnaporthe oryzae*, melanin formation and capsule production in *Cryptococcus neoformans*, and hyphal morphogenesis, adhesion, and invasion in *C*. *albicans*, among others [25–29].

Typically, fungi increase the environmental pH at a steady but slow pace. A clear exception is *C*. *albicans*, which is capable of ammonia-driven alkalinization at a remarkable rate. Upon growth on amino acids as the sole carbon source, this human pathogen can modulate the environmental pH from 4 to ~7.5 within few hours, a process also driven (albeit more slowly) by other *Candida* sp. [23, 30] The alkalinization mechanism has been studied extensively and includes sensing of amino acids from the extracellular milieu via the SPS (Ssy1, Ptr3, and Ssy5) sensor system, followed by activation of the transcription factor Stp2p in an SPS-dependent manner and induction of amino acid influx [31]. As a result, ammonia is generated and exported via ammonium transporters to raise the environmental pH and allow fungal transition to the more virulent hyphal form [32, 33]. Deletion of genes involved in any step of this mechanism leads to impaired generation of ammonia and neutralization of the medium in response to these nutrients. Thus, metabolism of amino acids is critical for pH modulation by this fungus.

*C*. *albicans* is closely associated with the host and has evolved to utilize a variety of nonpreferred carbon sources available in different anatomical sites. Metabolism of organic acids and N-acetylglucosamine also results in environmental alkalinization [23, 34, 35]. However, genes required for alkalinization on amino acids do not affect growth or pH changes on these nutrients, suggesting different mechanisms for pH modulation [35]. Most importantly, *C*. *albicans* cells grown on organic acids do not generate ammonia. How *C*. *albicans* generates a basic extracellular environment under these conditions is currently unknown. It is also not clear if other fungal species share the ability to neutralize the environment upon utilization of these nutrients.

## Regulation of ammonia production

Fungi regulate the production of ammonia depending on environmental cues. Ammonia production by *M*. *anisopliae* is tightly regulated by amino acids, a signal for the presence of proteinaceous nutrients in the environment. *M*. *anisopliae* grown in media containing low levels of single amino acids yields higher levels of ammonia than when amino acids are abundant, implying either induction of catabolite repressible enzyme(s) or regulation of enzyme activity via substrate inhibition [28]. Generation of ammonia by *N*. *crassa* and *A*. *fumigatus* is a loosely regulated process triggered by nutrient deprivation [36]. Presence of glucose in the environment represses the process, presumably due to the metabolic switch from gluconeogenesis to glycolysis or repression of deaminases and ammonia transporters. It is also possible that the higher growth rate in the presence of glucose allows for complete utilization of ammonia released from amino acid catabolism.

## Environmental alkalinization as a virulence factor

Many fungal pathogens modulate environmental pH as a means to escape host immune responses, facilitate destruction of the host tissues, and/or stimulate reproduction. Most fungi inhabit mildly acidic environments, such as soil, plant, and animal surfaces. On the other hand, for some fungi, such as the phytopathogens *C*. *gloeosporioides* and *M*. *oryzae*, acidic pH favors fungal colonization and invasion [12, 28]. The mildly acidic pH of the plant surface favors both germination of attached conidia and rapid differentiation of the germ tube into a specialized cell named appressorium. Once the appressorium penetrates the plant tissues, the fungus switches to necrotrophic development, associated with rapid ammonia release and increase in environmental pH, which triggers the expression of virulence factors [13, 22, 28, 37]. Thus, the acidic environment serves as a signal in this fungus to switch from saprotrophic to necrotrophic growth and damage the host (Fig 1).

**Fig 2:**
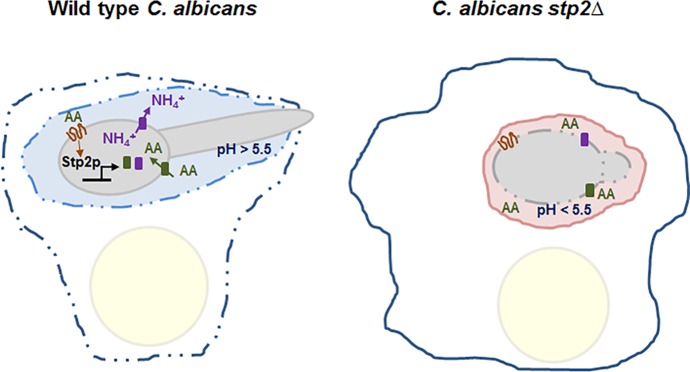
Neutralization of the macrophage phagosome by the fungal pathogen *C*. *albicans* is essential for host damage. Upon phagocytosis by the macrophages, *C*. *albicans* responds to the presence of amino acids and other alternative carbon sources abundant in the phagosomal milieu. Amino acids (AA) in particular are sensed via the SPS sensor system (orange), leading to activation of the transcription factor Stp2p, which induces the expression of genes encoding for amino acid permeases (green rectangles) and ammonium transporters (purple rectangles). This results in uptake of amino acids into and release of NH_4_^+^ from the fungal cell and increase of phagosomal pH. Hyphal growth causes physical damage to the macrophage membranes, leading to leakage of cellular content and death. *C*. *albicans* cells defective in utilization of amino acids and/or extrusion of NH_4_^+^, such as the *stp2Δ* mutant, fail to modulate the pH of the phagosome and are readily cleared by the immune cells.

Neutralization of acidic niches is a very common microbial strategy to evade host immune responses. For example, *C*. *albicans* neutralizes the macrophage phagosome, a process essential for germination and escape from the immune cell [31–33]. *C*. *albicans* genes essential for pH modulation in vitro fail to induce macrophage damage, highlighting the importance of this process in immune evasion (Fig 2) [31–33]. Similarly, *Candida glabrata* mutants with alkalinization defect in vitro, such as cells lacking functional mannosyltransferases, fail to effectively modify phagosomal pH and damage the macrophage [38]. The spherules of *Coccidioides* spp., causative agents of coccidioidomycosis, release enzymatically active urease and ammonia to raise the environmental pH and destroy the host tissue [39, 40]. Disruption of the urea degradation pathway in this organism significantly attenuates fungal virulence and survival in mice [39]. Thus, fungal pathogens can alkalinize the host environment to modulate their virulence, underlining the importance of this process on microbial and human physiology.

In summary, adaptation of fungi to pH variations in the host is critical for their survival, and fungal pathogens are capable of actively modulating the environmental pH. Acidification of the host tissues promotes expression and activity of fungal proteases. Many fungi utilize nitrogen or carbon metabolism pathways to generate ammonia, which is released from the cell to raise the extracellular pH. Generation of alkaline pH favors morphogenetic and reproductive processes in fungi, such as germination, hyphal growth, and formation of fruiting bodies, all critical for disease progression. The alkaline pH increases fungal virulence by facilitating penetration into host surfaces and hindering or evasion of immune responses. Thus, the ability to control extracellular pH is an important aspect of fungal physiology that contributes to fitness within the host.
